# Comparative Analysis of Amino Acid Profiles in Patients with Glioblastoma and Meningioma Using Liquid Chromatography Electrospray Ionization Tandem Mass Spectrometry (LC-ESI-MS/MS)

**DOI:** 10.3390/molecules28237699

**Published:** 2023-11-22

**Authors:** Piotr Kośliński, Robert Pluskota, Marcin Koba, Zygmunt Siedlecki, Maciej Śniegocki

**Affiliations:** 1 Department of Toxicology and Bromatology, Collegium Medicum in Bydgoszcz, Nicolaus Copernicus University in Toruń, dr. A. Jurasza 2, 85-089 Bydgoszcz, Poland; pluskota.r@gmail.com (R.P.); kobamar@cm.umk.pl (M.K.); 2 Department of Neurosurgery, Neurotraumatology and Pediatric Neurosurgery, Collegium Medicum in Bydgoszcz, Nicolaus Copernicus University in Toruń, 85-094 Bydgoszcz, Poland; siedlecki@cm.umk.pl (Z.S.); sniegocki@cm.umk.pl (M.Ś.)

**Keywords:** glioblastoma, meningioma, amino acid, biomarkers, LC-MS

## Abstract

Brain tumors account for 1% of all cancers diagnosed de novo. Due to the specificity of the anatomical area in which they grow, they can cause significant neurological disorders and lead to poor functional status and disability. Regardless of the results of biochemical markers of intracranial neoplasms, they are currently of no diagnostic significance. The aim of the study was to use LC-ESI-MS/MS in conjunction with multivariate statistical analyses to examine changes in amino acid metabolic profiles between patients with glioblastoma, meningioma, and a group of patients treated for osteoarthritis of the spine as a control group. Comparative analysis of amino acids between patients with glioblastoma, meningioma, and the control group allowed for the identification of statistically significant differences in the amino acid profile, including both exogenous and endogenous amino acids. The amino acids that showed statistically significant differences (lysine, histidine, α-aminoadipic acid, phenylalanine) were evaluated for diagnostic usefulness based on the ROC curve. The best results were obtained for phenylalanine. Classification trees were used to build a model allowing for the correct classification of patients into the study group (patients with glioblastoma multiforme) and the control group, in which cysteine turned out to be the most important amino acid in the decision-making algorithm. Our results indicate amino acids that may prove valuable, used alone or in combination, toward improving the diagnosis of patients with glioma and meningioma. To better assess the potential utility of these markers, their performance requires further validation in a larger cohort of samples.

## 1. Introduction

Intracranial neoplasms account for 2% of cancers, and deaths during these cancers account for 3% of all cancer deaths worldwide. Gliomas are among the most common CNS neoplasms and the most common primary malignant tumors. From a practical point of view, glial tumors are divided into low-grade gliomas (LGGs) and high-grade gliomas (HGGs) [1,2,3,4,5]. LGGs are diagnosed in the younger age group and grow slowly, but usually infiltratively. They are most often located within the temporal and frontal lobes and within the insula. The median survival time from diagnosis is seven years [6,7,8]. An HGG, in turn, is much more common in older people who are most often diagnosed with GBM (grade 4 according to the WHO). GBM is an aggressive tumor that grows dynamically and leads to a progressive decline in neurological performance. The average survival of patients with an HGG is 16 to 36 months [4,5,9].

Meningiomas are the most common primary intracranial neoplasms. Most often, meningiomas are solitary intracranial tumors that arise without obvious risk factors. However, possible factors contributing to the development of meningiomas include radiation therapy, inflammation, intracranial trauma, and hormonal disorders [10,11,12].

Clinical symptoms of intracranial tumors have a twofold pathophysiological basis. Firstly, they result from increased intracranial pressure (ICP) and are non-specific, and secondly, they are related to the location of the lesion and depend on the neural centers and tracts occupied or compressed by the tumor. The most common symptom of all intracranial tumors is progressive neurological deficit [13,14].

The emergence and development of intracranial tumors are conditioned by genetic, molecular, cellular, and biochemical mechanisms. As in the case of any carcinogenesis, including CNS tumors, their growth is a multi-stage process underpinned by genetic mutations [15].

Imaging tests (MRI, CT) are the basic method of monitoring the course and treatment of brain tumors, while biochemical markers determined in urine and body fluids have limited use so far. Previous studies indicate the possibility of using various compounds as potential biomarkers in CNS tumors, such as interleukins [16,17], the S100 Protein Superfamily [18,19,20,21,22], proteins reacting in the acute phase and other markers of inflammatory proteins [19,22,23,24,25], and panels of peptides and proteins [24,26,27]. Despite many studies, none of the biomarkers are ready for direct clinical implementation. A key criterion for the potential clinical value of a candidate cancer biomarker is the consistency and strength of the association between the biomarker and the treatment outcome or disease of interest and the extent to which this represents a diagnostic improvement over methods already in use. The intensity of work on potential biomarkers of CNS tumors is reflected in the number of scientific works, the summary and critical analysis of which can be found in review works [28,29,30].

Metabolomics is a complementary approach to genomics and proteomics focused on the comprehensive profiling of all small molecular weight metabolites in biofluids, tissues, and cells. Metabolomics approaches can be targeted or non-targeted. Untargeted metabolomics focuses on the unbiased, hypothesis-free analysis of all detectable metabolites from a biological sample. Although this approach allows for the identification of a large pool of potentially relevant metabolites, the approach often represents a trade-off between specificity, selectivity, and analysis time. Targeted metabolomics refers to the quantitative measurement of a select group of metabolites (e.g., amino acids) in order to investigate specific metabolic pathways or validate biomarkers identified using non-targeted metabolic profiling. Once a potentially relevant metabolic pathway has been identified using an untargeted approach, targeted metabolic profiling appears advisable to increase the number of metabolites associated with that particular pathway [31,32].

A wide range of analytical techniques are available for the qualitative and quantitative determination of metabolites; however, the most commonly used analytical techniques in cancer metabolomics are MS and NMR spectroscopy.

NMR spectroscopy is a quantitative, high-throughput technology that provides both qualitative and quantitative information, making it a useful tool in metabolomics research. Despite the many advantages of NMR spectroscopy, due to its relatively low sensitivity and poor dynamic range, it is not suitable for the detection of metabolites present in trace amounts. Most metabolomics studies of brain tumors are based on NMR spectroscopy, which allows for the observation of changes in metabolism related to tumor development and differentiation of the degree of malignancy [31,32,33,34,35].

Mass spectrometry (MS) provides a valuable analytical platform for metabolomics due to its high sensitivity and wide dynamic range. With the dissemination of this technology to routine medical laboratories, it has become possible to measure low molecular weight metabolites for diagnostic purposes. The use of LC-MS/MS methods for small molecules in biological fluids is becoming increasingly preferred because they have higher sensitivity and better reliability in complex biological fluids. Recent studies have shown that the use of metabolomic analysis with mass spectrometry detection allowed the identification of biomarkers differentiating patients with CNS tumors from healthy people or enabling the differentiation of low- and high-grade tumors [34,36,37,38,39,40,41].

There are several ways to assess changes in the metabolome in patients with CNS tumors: through the metabolome of plasma collected from an antecubital vein or the metabolomic analysis of blood collected from resected tumor tissue or cerebrospinal fluid [31,32,38,39,40,42]. Griffin et al.’s review summarizes progress in the field of human brain tumor metabolomics research using tissue sections using both ex vivo and in vivo approaches [43]. Another approach to assessing changes in metabolism was presented in the work of Xiong et al., who used the method of collecting intraoperative blood samples from the artery located directly above the brain tumor and the vein located directly behind the brain tumor [44].

Amino acids (AAs) are the building blocks of various biomacromolecules, as well as functional regulators of various metabolic processes. The altered metabolism of AAs promotes the proliferation of tumors and contribute to their immune escape and resistance to treatment. From a clinical point of view, it is estimated that changes in amino acid concentrations observed in specific body fluids provide the opportunity to develop new diagnostic and prognostic markers [45,46]. Early diagnostic blood markers are biomarkers that can be used to predict the development of cancer in an individual, many years before clinical or radiological signs are noticed. These markers can be useful for screening patients at risk. A prognostic biomarker is defined as a clinical or biological characteristic that provides information on the most probable patient health outcome irrespective of the treatment [29].

The aim of the study is to evaluate the potential of targeted dual-control serum amino acid metabolomics analysis in the diagnosis of patients with glioma and meningioma. In pilot studies, other groups have explored the use of a metabolomics approach to distinguish patients with glioblastoma and meningioma from healthy controls. However, to the best of our knowledge, no metabolomics study based on targeted amino acid analysis has attempted to remove the confounding effects of common pathophysiology, such as a generalized immune response.

The present study used high-performance liquid chromatography–mass spectrometry (HPLC-MS) coupled with multivariate statistical analyses to accurately identify unique changes in amino acid metabolomic profiles that distinguish glioma patients from meningioma patients and other diseases that may be associated with overall inflammation, e.g., degenerative spine disease.

## 2. Results

### 2.1. Comparison of the Control Group and Cancer Patients

**Figure 1:**
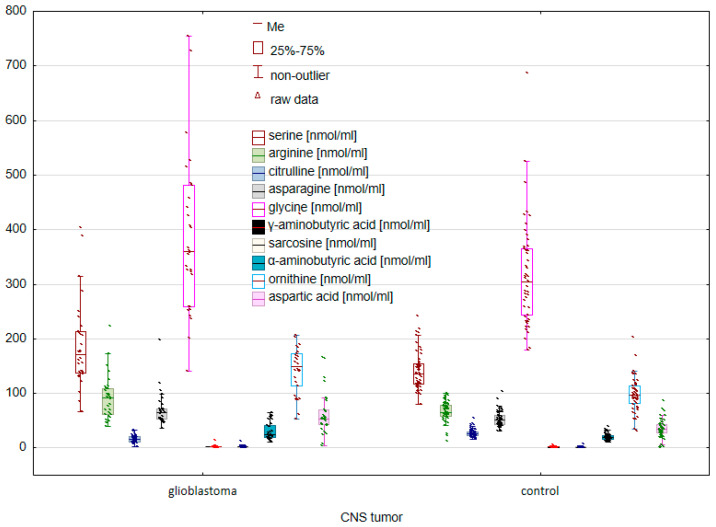
Comparison of endogenous AAs between controls and cancer patients with glioblastoma.

**Figure 2:**
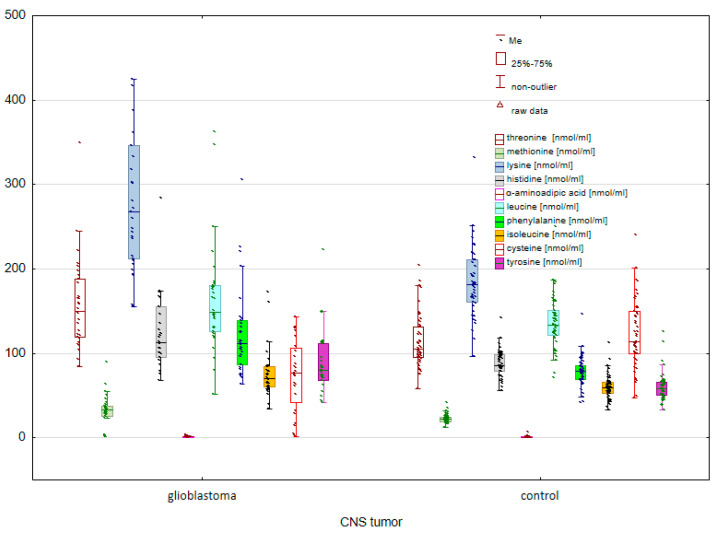
Comparison of exogenous AAs between controls and cancer patients with glioblastoma.

The first stage of the study was to compare amino acid concentrations between patients diagnosed with glioblastoma and the control group. A comparative analysis of the analyzed AAs between patients with glioblastoma and the control group allowed the identification of statistically significant differences between the study groups. Statistically significant differences were observed both among endogenous (Figure 1) and exogenous (Figure 2) amino acids.

Among endogenous AAs, statistically significant differences concerned SER (*p* = 0.002245), ARG (*p* = 0.009292), CIT (*p* = 0.000000), ASN (*p* = 0.000939), GLY (*p* = 0.017846), GABA (*p* = 0.000000), SAR (*p* = 0.019362), ABA (*p* = 0.001831), ORN (*p* = 0.000012), and ASP (*p* = 0.000214).

In the case of exogenous amino acids, statistically significant differences were observed for THR (*p* = 0.000032), MET (*p* = 0.000190), LYS (*p* = 0.000000), HIS (*p* = 0.000011), AAA (*p* = 0.000021), LEU (*p* = 0.036077), PHE (*p* = 0.000001), ILE (*p* = 0.000728), C-C (*p* = 0.000037), and TYR (*p* = 0.000007).

**Figure 3:**
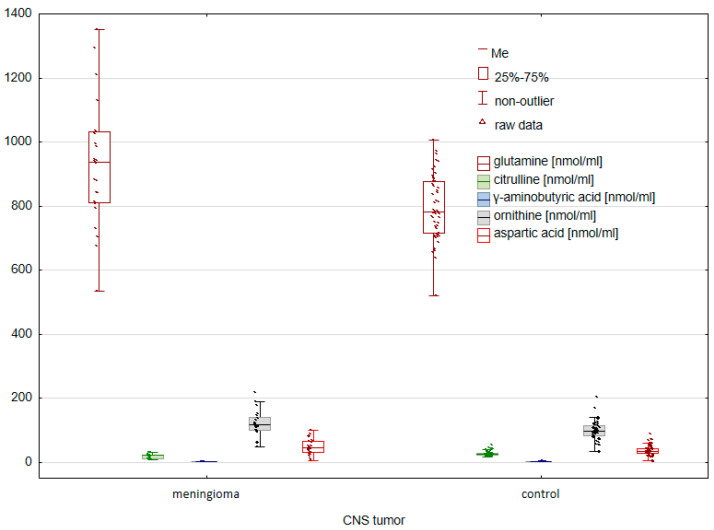
Comparison of endogenous AAs between controls and cancer patients with meningioma.

**Figure 4:**
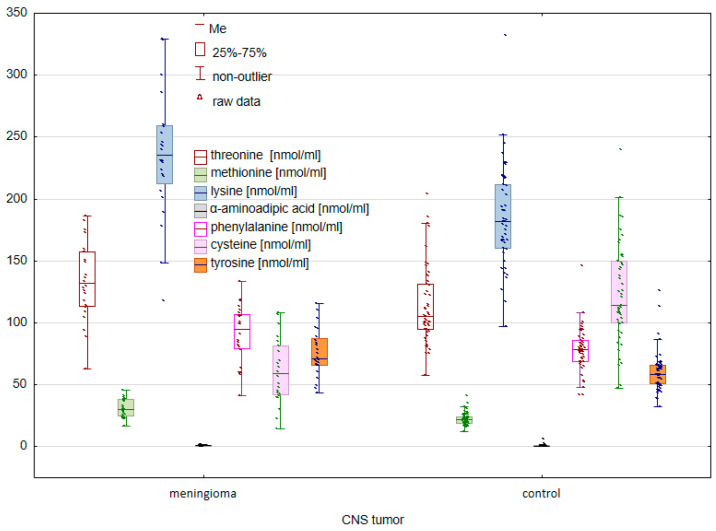
Comparison of exogenous AAs between controls and cancer patients with meningioma.

In the next stage, the concentrations of the tested amino acids were compared between the group of patients with meningioma and the control group. The U Mann–Whitney U test allowed us to identify statistically significant differences between the study groups (Figure 3 and Figure 4).

In the case of endogenous amino acids, significant differences were found for GLN (*p* = 0.001167), CIT (*p* = 0.000028), GABA (*p* = 0.000145), ORN (*p* = 0.005893), and ASP (*p* = 0.049001). A comparison of the group of patients with glioma and the control group also allowed for the confirmation of statistically significant differences for such exogenous amino acids such as THR (*p* = 0.005100), MET (*p* = 0.000003), LYS (*p* = 0.000019), AAA (*p* = 0.007040), PHE (*p* = 0.007552), C-C (*p* = 0.000000), and TYR (*p* = 0.000349).

To eliminate the influence of the variability related to the sex of the studied groups on the observed differences in amino acid concentrations, a comparison was made taking into account the division by sex. The obtained results made it possible to isolate those amino acids for which statistically significant differences were found between the study group of cancer patients and the control group, both in the group of women and men.

**Figure 5:**
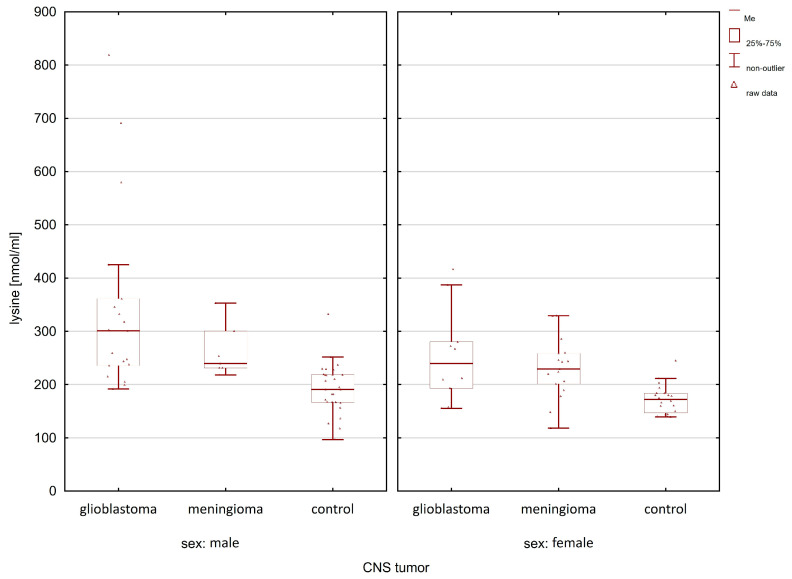
Comparison of lysine concentrations between glioma patients, meningioma patients, and the control group in the male and female group.

**Figure 6:**
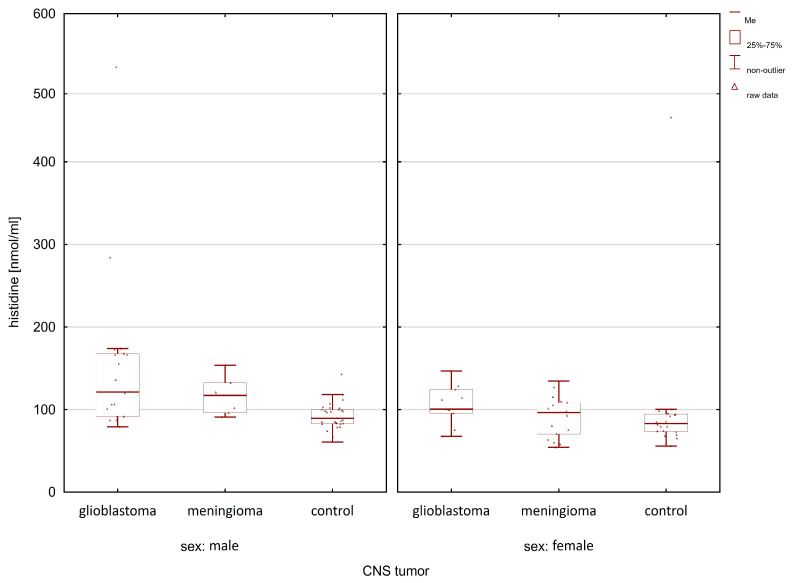
Comparison of histidine concentrations between glioma patients, meningioma patients, and the control group in the male and female group.

**Figure 7:**
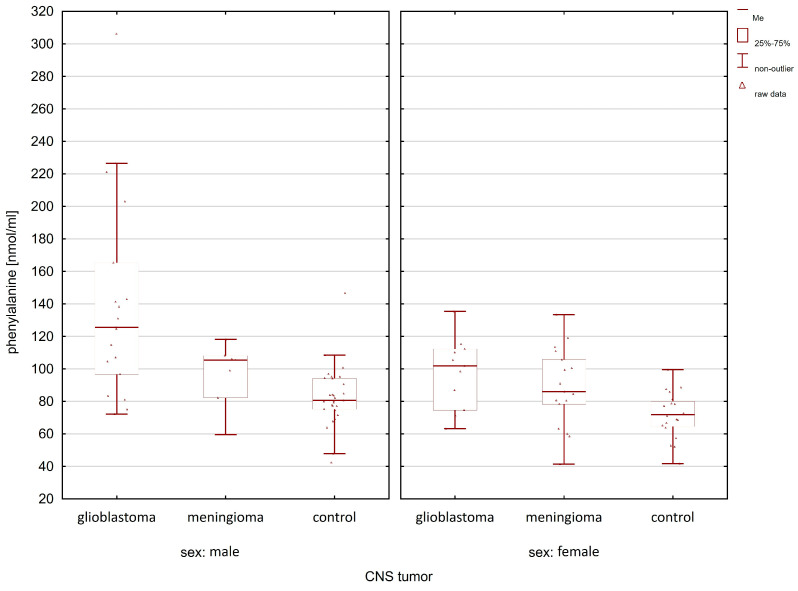
Comparison of phenylalanine concentrations between glioma patients, meningioma patients, and the control group in the male and female group.

**Figure 8:**
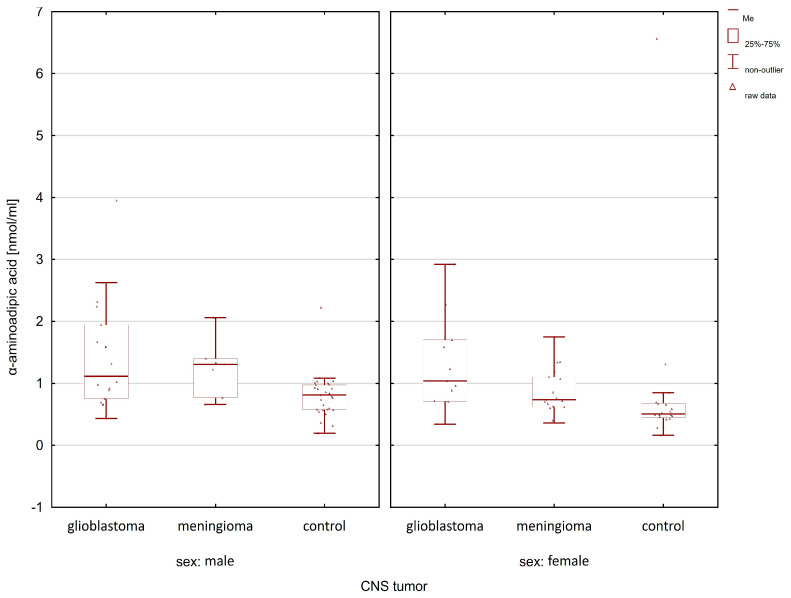
Comparison of α-aminoadipic acid concentrations between glioma patients, meningioma patients, and the control group in the male and female group.

When comparing the group of patients with glioblastoma with the control group, statistically significant differences after post hoc analysis were observed for lysine, histidine, α-aminoadipic acid, and phenylalanine (Table 1). In the case of meningioma patients, compared to the control group, statistically significant differences in the group of women and men were observed only for lysine (women *p*-value = 0.0032; men *p*-value = 0.0120). Figure 5, Figure 6, Figure 7 and Figure 8 illustrate the observed differences for amino acids with statistically significant differences, both in the group of men and women.

In Figure 5, the highest lysine concentration can be observed in glioma patients compared to the controls and meningioma patients. Similar relationships of amino acid concentrations (i.e., the highest in patients with glioma and the lowest in the control group) between the studied groups can be observed in the case of histidine (Figure 6) and phenylalanine (Figure 7).

Only in the case of α-aminoadipic acid was the concentration of this amino acid higher in the group of men in patients with meningioma compared to the group with glioma and the control group (Figure 8).

### 2.2. Classification Analysis

#### 2.2.1. Receiver Operating Characteristic Curves

**Figure 9:**
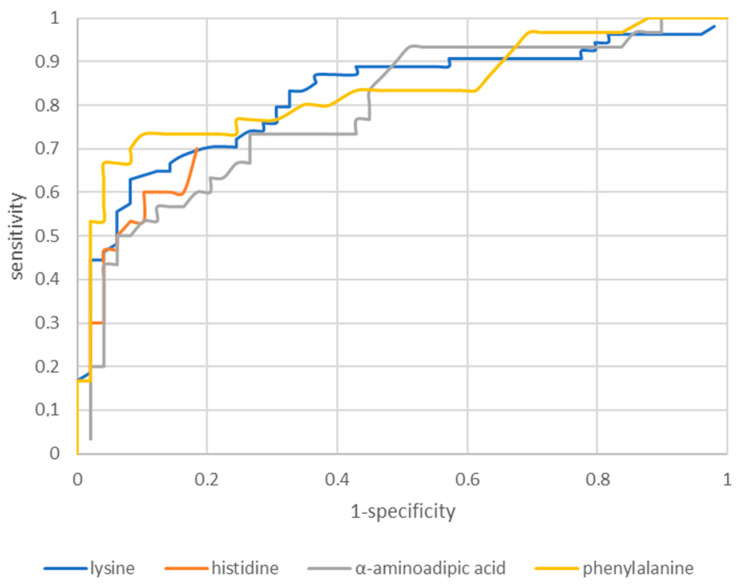
Visualizations of sensitivity and the specific model of ROC analysis.

The next part of the analysis started to build a classification model that will classify patients as healthy or suffering from glioblastoma of the CNS with the highest efficiency. Receiver operating characteristic (ROC) analysis is a useful tool for evaluating the accuracy of a model’s prediction by plotting the sensitivity versus (1-specificity) a classification test. The model of the classification of patients into the group with glioblastoma and the control group included those AAs in which statistically significant differences were found between the study group and the control group, both in the group of women and men. Visualizations of the obtained results are presented in Figure 9.

**Figure 10:**
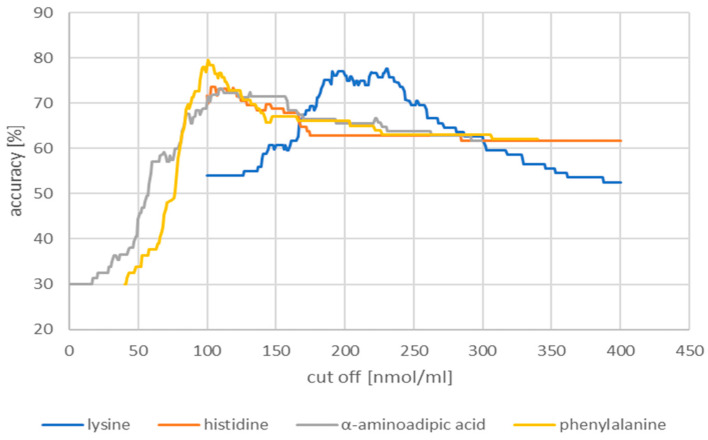
Visualizations of the accuracy model of ROC analysis.

Then, the accuracy of the model was assessed in relation to the concentration of the analyzed amino acids. The obtained results are illustrated in Figure 10.

The obtained cut-off, accuracy, sensitivity, and specificity values of the tested amino acids obtained using the ROC curve are summarized in Table 2.

#### 2.2.2. Decision Trees

**Figure 11:**
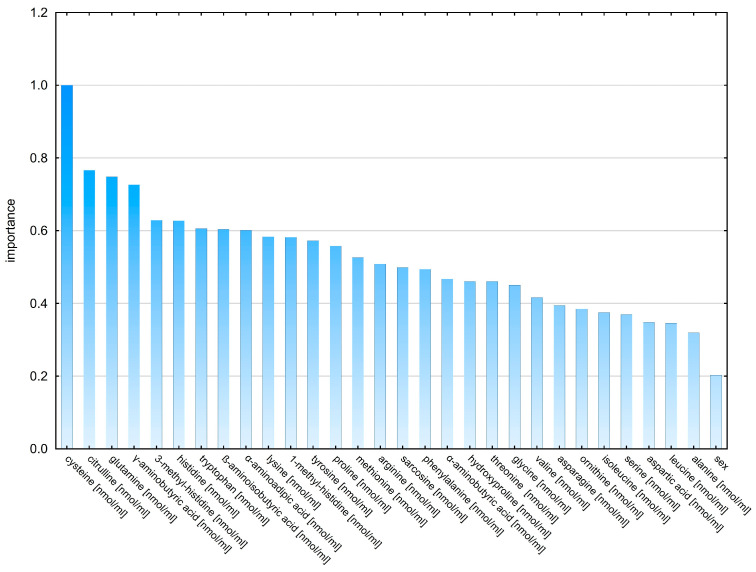
Graph of the validity of model variables based on aggregated data.

The analysis was aimed at showing which amino acids would allow for a better classification of patients into a group with glioblastoma and a control group of healthy people. Classification trees are used to build predictive models (predicting subsequent data based on the model based on previously provided information) and descriptive models. The significance plot of the model variables based on standardized data primarily indicates the concentration of cysteine, citrulline, and glutamine (Figure 11).

**Figure 12:**
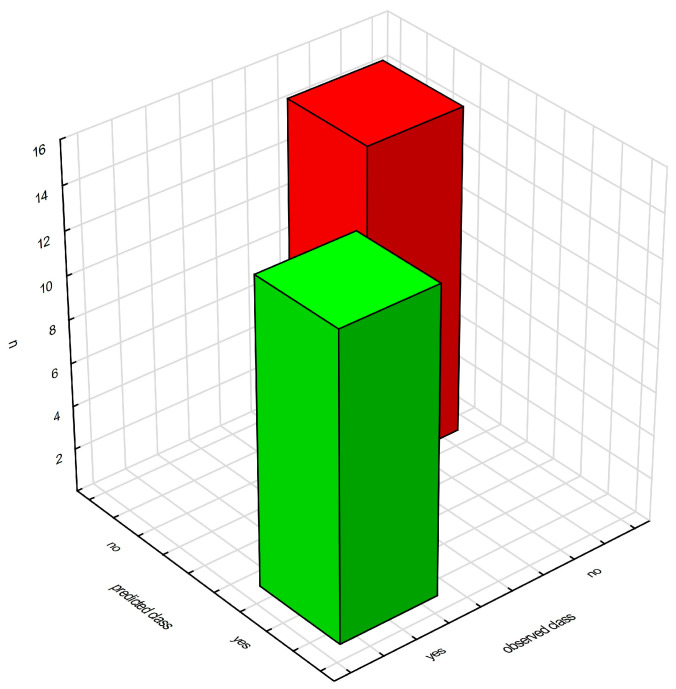
Matrix of patient classification made by a model based on aggregated data. Green—glioma patients correctly classified. Red—control group classified as healthy.

Importantly, the use of a model based on the above parameters gives a much better chance of correctly diagnosing a person with glioblastoma than diagnosing the same cancer in a healthy person (Figure 12).

## 3. Discussion

Many research projects focus on finding prognostic or diagnostic biomarkers for central nervous system tumors, including meningiomas and gliomas. Currently, classical metabolic pathways such as the citric acid cycle (TCA), glycolysis, and arachidonic acid/inflammatory pathway have been well-studied in glioma. However, in recent years, attention has also been paid to understanding new metabolic changes in glioma cells, such as metabolic changes in IDH mutant cells, amino acid metabolism, or nucleotide metabolism. Many characteristic metabolites have been discovered in glioma, such as 2-HG, fumaric acid, succinic acid, sarcosine, glycine, glutamine, aspartic acid, choline, serine, glucose, lactic acid, and polyamines, which suggest the dysregulation of multiple metabolic pathways (e.g., glycolysis, TCA, glutaminolysis, pentose phosphate pathway, fatty acid metabolism, amino acid metabolism) [34,36,37,47,48,49,50,51].

This study was designed to evaluate changes in amino acid profiles between patients with glioblastoma, meningioma, and the control group. Available studies indicate that dysregulated AA metabolism is not characteristic of a given type of cancer. The selection of the study groups (glioblastoma vs. meninges vs. a group of patients treated for osteoarthritis of the spine as a control group) was aimed at capturing possible differences in the profiles of the tested amino acids, depending on the type of cancer.

The conducted analysis allowed the observation of statistically significant differences between the study groups. When we compare the changes in the amino acid profiles of the glioblastoma and meningioma patients to the controls, we can see a group of amino acids that differ in both cases. This applies to endogenous amino acids (CIT, GABA, ORN, ASP) and exogenous (THR, MET, LYS, AAA, PHE, C-C, and TYR). In the case of glioblastoma, we observe additional amino acids differentiating the study group and the control group, including SER, ARG, ASN, GLY, SAR, and ABA. This points to a more altered amino acid metabolism in the glioblastoma group. In the case of meningioma, attention is paid to glutamine, whose elevated concentration is observed in comparison with the control group. In patients with glioblastoma, these differences are not statistically significant. In most cases, we observe a higher concentration of amino acids in groups of cancer patients compared to the control group. Lower concentrations were observed with cysteine, the concentration of which is statistically significantly lower in patients with glioblastoma compared to the control group. In turn, the concentration of cysteine is reduced in both glioblastoma and meningioma patients compared to the control group.

The biomarker should be resistant to inter- and intra-individual factors, such as gender. Therefore, in the proposed study, the study groups of both cancer patients and the control group were divided by gender. Among the compounds that showed statistically significant differences, only lysine, histidine, α-aminoadipic acid, and phenylalanine differentiated patients with glioblastoma from healthy ones both in the group of men and women. In patients with meningioma, differences in the group of men and women concerned only lysine.

The AAs that showed statistically significant differences were evaluated for diagnostic usefulness. The diagnostic usefulness of AAs determined in the identification of glioblastoma was assessed based on the ROC curve with the proposed cut-off point and area under the AUC curve. Sensitivity, specificity, and accuracy are given for the designated cut-off points. The best results were obtained for phenylalanine. The ROC graph for phenylalanine showed a sensitivity of 66.7%, a specificity of 95.9%, and an accuracy of 79.5% for the proposed cut-off of 101.00 nmol/mL. The other analyzed amino acids were characterized by similar parameters of lysine (cut-off point 230 nmol/mL, sensitivity 77.7%, specificity 63%, accuracy 91.8%), histidine (cut-off point 103 nmol/mL, sensitivity 73.7%, specificity 60%, accuracy 89.8%), and α-aminoadipic acid (cut-off 1.09 nmol/mL, sensitivity 73.2%, specificity 50%, accuracy 91.8%).

In the next stage of the research, classification trees were used, which allowed us to build a model allowing for the correct classification of patients into the study group (patients with glioblastoma) and the control group (healthy individuals) (Figure 11). Cysteine turned out to be the most important amino acid in the decision-making algorithm, followed by a group of three amino acids (citrulline, glutamine, γ-aminobutyric acid).

Analyzing the results of our research, it seems that the most important amino acids in terms of diagnostic decisions are lysine, aminoadipic acid, histidine, and phenylalanine. The higher concentration of lysine observed in our study in patients with glioma and meningioma, considering the division by gender, was also observed in patients with head and neck cancers and sarcoma [52,53,54]. Toklu et al. found high levels of serum lysine in high-grade glioma patients [55]. Mören et al. observed that serum lysine levels of high-grade glioma patients decreased during radiotherapy when the disease is indolent [56]. It is worth noting that in patients with glioblastoma, we also found statistically significantly higher concentrations of aminoadipic acid, which is a poorly characterized product of lysine degradation and may appear in the circulation because of the degradation of whole tissue or plasma proteins. The ε-amino group of lysine residues in proteins can undergo deamination to form an intermediate lysine, which during subsequent oxidation forms aminoadipic acid. Available data indicate that plasma aminoadipic acid plays a role in the modulation of glucose levels through a compensatory response to hyperglycemia, resulting in increased insulin secretion in early insulin resistance [57]. Cadoni et al. noted a high level of aminoadipic acid in the serum of patients with a reduced risk of advanced head and neck cancer [54]. Toklu et al. did not observe statistically significant differences in aminoadipic acid between high-grade glioma and the control group determined in serum and brain tissue [55].

We found high levels of serum histidine in glioblastoma patients compared to the controls. This was parallel with the result of Toklu et al., where histidine levels were elevated in patients with high-grade glioblastoma [55]. Previous studies found that low plasma levels of histidine were observed in patients with shorter overall survival in glioblastoma patients. The potential role of histidine in the neoplastic process is not clear. It is believed that some AAs seemingly unrelated to the cancer process are used to increase the rate of uptake of other AAs. The upregulation of amino acid transporters in cancer maintains amino acid pools at levels that support its malignant characteristics. This can be highlighted by several examples: the export of glutamine to drive leucine uptake, asparagine for serine, arginine, and histidine, and glutamate for cystine [58].

The results of our study indicate an altered metabolism of phenylalanine in both glioblastoma and meningioma patients. In addition, we showed the statistical significance of phenylalanine differentiating patients with glioblastoma and the control group, considering a division by gender. Phenylalanine was also characterized by the best diagnostic values in the case of patients with glioblastoma. Phenylalanine is the precursor of tyrosine, an important AA for the biosynthesis of neurotransmitters like L-DOPA (L-3,4-dihydroxyphenylalanine) and catecholamines dopamine, epinephrine, and norepinephrine. Available research results indicate an increased concentration of phenylalanine in cancer, which reflects increased muscle proteolysis. For example, elevated levels of phenylalanine have been observed in squamous cell carcinoma of the head and neck [54] and hepatocellular carcinoma [59]. In a study comparing metabolites produced and consumed by glioma, Xiong et al. indicated phenylalanine as one of the metabolites intensively consumed by glioma cells [44]. The reason for the increase in phenylalanine levels in cancer is not fully understood. It is believed to be related to inflammation, the activation of the immune system, and increased levels of markers of immune activation, including tumor necrosis factor (STNF R75) and neopterin [60]. Phenylalanine is required to produce tyrosine. This conversion is catalyzed by the enzyme phenylalanine hydroxylase (EC 1.14.16.1), which functions primarily in the liver but has been shown to be active in other tissues of the body, including the brain. It has also been observed that phenylalanine hydroxylase activity may be sensitive to inflammation or cancer [61,62].

## 4. Materials and Methods

### 4.1. Subjects and Serum Samples

This study included 54 patients treated surgically for intracranial tumors in the Department of Neurosurgery, Neurotraumatology, and Paediatric Neurosurgery at the University Hospital No. 1, Collegium Medicum of the Nicolaus Copernicus University. The histological types of tumors in the examined patients were glial tumors and meningiomas. Table 3 shows the clinical and demographic characteristics of the study group.

The control group consisted of 49 people (29 men, 20 women) treated for osteoarthritis of the spine. The exclusion criteria for the control group were past or current neoplastic disease and (similarly to the study group) a surgical procedure performed less than 30 days ago. This study was conducted in accordance with all internationally approved human testing guidelines and was in accordance with the Declaration of Helsinki. The Ethics Committee of the Nicolaus Copernicus University in Toruń and Collegium Medicum in Bydgoszcz approved this study (consent number KB 499/2021).

### 4.2. Sample Preparation

Antecubital whole-blood samples were drawn from a peripheral vein in the morning hours (always between 7 and 8 a.m.). Overnight fasting and 15 min of rest before the blood test were obligatory. Blood collected in the tube was kept for 30 min. Serum from the blood after clotting was separated, centrifuged, and frozen at −80 °C until analysis was performed. The EZ:faast amino acid analysis kit was utilized for serum sample preparation.

The EZ:faast amino acid analysis procedure consists of a solid phase extraction step followed by derivatization and a liquid/liquid extraction. The solid phase extraction is performed via a sorbent-packed tip that binds amino acids while allowing interfering compounds to flow through. Amino acids on sorbent are then extruded into the sample vial and quickly derivatized with a reagent at room temperature in an aqueous solution. Derivatized amino acids concomitantly migrate to the organic layer for additional separation from interfering compounds. The organic layer is then removed, evaporated, and re-dissolved in an aqueous mobile phase and analyzed on an LC-MS system.

### 4.3. Instrumentation and Conditions

The quantitative and qualitative analyses of samples were performed using High-Performance Liquid Chromatograph Nexera XR LC-20 AD pump (Shimadzu, Kyoto, Japan) and a Nexera XR SIL-20AC autosampler (Shimadzu, Kyoto, Japan) coupled with a mass spectrometer equipped with an electrospray ion source (ESI), an LCMS-8045 Mass Spectrometer (Shimadzu, Kyoto, Japan). The instrument was controlled, and recorded data were processed using LabSolutions LCMS Ver.5.6 software.

Chromatographic separation was carried on the EZ:faast amino acid analysis–mass spectrometry column (250 × 3.0 mm, 4 µm) at a column temperature of 35 °C with the corresponding binary mobile phase. Solvent A was 10 mM ammonium formate in water and solvent B was 10 mM ammonium formate in methanol. The mobile phase flow was 0.25 mL/min and took place in a gradient system: 68% B–83% B in 13 min. The injection volume was 1 µL. The mass spectrometry (MS) data were acquired in positive ion mode. Multiple reacting monitoring was used for quantification by monitoring the ion transition of amino acids.

Selected mass spectrometry parameters are summarized in Table 4.

### 4.4. Reagents and Solvents

The EZ:faast^TM^ amino acid analysis kit was obtained from Phenomenex, Inc. (Torrance, CA, USA). HPLC grade methanol was obtained from Merck (Darmstadt, Germany). Ammonium acetate and formic acid of analytical grade were purchased from Sigma-Aldrich Co. (St. Louis, MO, USA) with purity greater than 99%. Water was deionized and purified using a Milli-Q system (Millipore, Bedford, MA, USA) and used to prepare all aqueous solutions.

Calibration curves for each AA were constructed in a range from 0.01 to 75 nmol/mL. Calculation and calibration are based on an internal standard method. The kit contains as an internal standard an AA mixture of homoarginine (HARG), methionine-d3 (Met-d3), and homophenylalanine (HPHE).

### 4.5. Statistical Analysis

The statistical analysis was performed using Statistica 13.1 (StatSoft) and Excel 365 (Microsoft). The type of distribution was examined using the Shapiro–Wilk test. The Levene test was used to test the homogeneity of variance. The Kruskal–Wallis test and multiple comparisons of mean ranks for all trials were used to assess differences between more than two groups. Relationships between variables were described using the Spearman coefficient in the event of statistically significant differences. The data were visualized using a box plot. Principal component analysis (PCA) was used to classify correlation coefficients. The correlation matrix was used as the input. To isolate the number of principal components, Cattella’s and Kaiser’s criteria were used.

The receiver operating characteristic (ROC) curves were prepared using the concentration values of amino acid compounds. Sensitivity (SEN), specificity (SPE), and accuracy (Acc) were used to evaluate the model. The general models of regression and classification trees were used to build the classification model. The collected data were divided by a 4:1 ratio into a learning and testing group. Cross-validation was used to verify the effectiveness of the models. All analyses were performed at a significance level of 5% (α = 0.05).

## 5. Conclusions

A comparative analysis of AAs between patients with glioblastoma, meningioma, and the control group allowed for the identification of statistically significant differences between the study groups.

A comparison of the amino acid profile between patients with glioma and the control group allowed for the identification of differences in both endogenous amino acids (SER, ARG, CIT, ASN, GLY, GABA, SAR, ABA, ORN, ASP) and exogenous amino acids (THR, MET, LYS, HIS, AAA, LEU, PHE, ILE, CC, TYR). Similarly, when comparing the amino acid profiles of patients with meningioma to the control group, the differences observed concerned endogenous amino acids (GLN, CIT, GABA, ORN, ASP) and exogenous amino acids (THR, MET, LYS, AAA, PHE, C-C, TYR). When comparing the amino acid profile by gender, only lysine showed statistically significantly higher concentrations in the group of patients with glioma and in the group of patients with meningioma compared to the control group. In the group of glioma patients, differences in amino acid profiles by gender also concerned histidine, α-aminoadipic acid, and phenylalanine. However, no statistically significant differences were observed when comparing amino acid profiles between the groups of patients with glioma and meningioma. The AAs that showed statistically significant differences were evaluated for diagnostic usefulness. The best results were obtained for phenylalanine.

This study showed that amino acids may be involved in the mechanisms underlying the pathogenesis of glioblastoma and meningioma and may also be considered potential indicators of some pathways altered in CNS neoplastic diseases. However, given the multifactorial, heterogeneous, and complex nature of these diseases, further work with a larger study group is needed to correlate the results of previous studies with possible other malignancies to see if the amino acid profile of meningiomas and gliomas can be considered characteristic of these diseases.

## Figures and Tables

**Table 1:** Statistically significant differences between cancer group glioblastoma and the control in with regard to sex.

Compounds	Women *p*-Value	Men *p*-Value
lysine	0.0059	0.0000
histidine	0.0250	0.0004
α-aminoadipic acid	0.0026	0.0060
phenylalanine	0.0088	0.0001

**Table 2:** Classification parameters of a model based on the ROC.

	Cut-off (nmol/mL)	Accuracy (%)	Sensitivity (%)	Specificity (%)
Lysine	230.00	77.7	63.0	91.8
Histidine	103.00	73.7	60.0	89.8
α-aminoadipic acid	1.09	73.2	50.0	91.8
Phenylalanine	101.00	79.5	66.7	95.9

**Table 3:** Clinical and demographic characteristics of the study group.

CNS Tumor	Glioma (*n* = 30)	Meningioma (*n* = 24)
**Age**	62.5	59.6
**Sex**		
- Female	11 (36.67%)	17 (70.83%)
- Male	19 (63.33%)	7 (29.17%)
**WHO Classification**		
- HGG (4. WHO)	30	
- Meningothelial meningiomas (1. WHO)		13
- Transitional meningiomas (1. WHO)		3
- Fibroblastic meningiomas (1. WHO)		3
- Atypical meningiomas (2. WHO)		5

**Table 4:** Amino acids, AA abbreviated name, retention time (min.), transitions chosen for each compound.

Compound Name	Abbreviated Name	t_R_ (min)	Quantification Transition
Serine	SER	3.885	233.9 > 146.00
Glutamine	GLN	3.468	275.1 > 172.00
Arginine	ARG	3.557	303.1 > 69.00
Citrulline	CIT	3.549	304.1 > 156.00
Homoarginine	HARG (IS)	3.753	317.10 > 84.00
Asparagine	ASN	3.985	243.10 > 157.00
1-Methyl-l-histidine	1MHIS	4.201	298.1 > 96.00
3-Methyl-l-histidine	3MHIS	4.235	298.1 > 210.00
4-Hydroxyproline	HYP	4.183	260.1 > 172.1
Glycine	GLY	4.423	203.9 > 144.00
Threonine	THR	4.482	248.10 > 160.00
Alanine	ALA	5.347	218.00 > 130.00
Gamma-aminobutyric acid	GABA	5.739	232.00 > 172.00
Sarcosine	SAR	5.948	217.90 > 88.00
Beta-aminoisobutyric acid	BAIB	6.202	232.00 > 172.00
α-Aminobutyric acid	ABA	6.567	232.00 > 172.00
Ornithine	ORN	6.795	347.00 > 287.00
Methionine	MET	7.085	227.9 > 190.10
Methionine-d3	Met-d3 (IS)	7.030	281.1 > 193.00
Proline	PRO	7.130	244.00 > 156.00
Lysine	LYS	7.743	361.00 > 301.10
Aspartic acid	ASP	7.753	304.00 > 216.10
Histidine	HIS	7.826	370.10 > 196.00
Valine	VAL	8.116	246.00 > 158.00
Glutamic acid	GLU	8.223	317.50 > 172.10
Tryptophan	TRP	8.468	333.10 > 245.10
α-Aminoadipic acid	AAA	9.202	332.00 > 244.10
Leucine	LEU	9.593	260.00 > 172.10
Phenylalanine	PHE	9.679	294.10 > 206.10
Isoleucine	ILE	9.997	260.00 > 172.10
Homophenylalanine	HPHE (IS)	11.224	308.00 > 220.00
Cystine	C-C	11.402	497.00 > 248.00
Tyrosine	TYR	11.999	395.90 > 136.00

## Data Availability

Data are unavailable due to privacy or ethical restrictions.
